# Biomaterial Applications in Cell-Based Therapy in Experimental Stroke

**DOI:** 10.1155/2016/6810562

**Published:** 2016-05-04

**Authors:** Ligia S. B. Boisserand, Tomonobu Kodama, Jérémie Papassin, Rachel Auzely, Anaïck Moisan, Claire Rome, Olivier Detante

**Affiliations:** ^1^Inserm, U1216, BP 170, 38042 Grenoble Cedex 9, France; ^2^Grenoble Institut des Neurosciences (GIN), Université Grenoble Alpes, 38000 Grenoble, France; ^3^CAPES Foundation, Ministry of Education of Brazil, 70040-020 Brasília, DF, Brazil; ^4^Institute for Frontier Medical Sciences, Department of Reparative Materials, Kyoto University, Kyoto 606-8507, Japan; ^5^CHU Grenoble Alpes, Stroke Unit, Department of Neurology, CS 10217, 38043 Grenoble, France; ^6^CERMAV, CNRS, CERMAV, Université Grenoble Alpes, 38000 Grenoble, France; ^7^Cell Therapy and Engineering Unit, EFS Rhône Alpes, 464 route de Lancey, 38330 Saint-Ismier, France

## Abstract

Stroke is an important health issue corresponding to the second cause of mortality and first cause of severe disability with no effective treatments after the first hours of onset. Regenerative approaches such as cell therapy provide an increase in endogenous brain structural plasticity but they are not enough to promote a complete recovery. Tissue engineering has recently aroused a major interesting development of biomaterials for use into the central nervous system. Many biomaterials have been engineered based on natural compounds, synthetic compounds, or a mix of both with the aim of providing polymers with specific properties. The mechanical properties of biomaterials can be exquisitely regulated forming polymers with different stiffness, modifiable physical state that polymerizes* in situ, *or small particles encapsulating cells or growth factors. The choice of biomaterial compounds should be adapted for the different applications, structure target, and delay of administration. Biocompatibilities with embedded cells and with the host tissue and biodegradation rate must be considerate. In this paper, we review the different applications of biomaterials combined with cell therapy in ischemic stroke and we explore specific features such as choice of biomaterial compounds and physical and mechanical properties concerning the recent studies in experimental stroke.

## 1. Introduction

The stroke is a major public health issue in the world due to aging populations and the socioeconomic burden of neurovascular disorders. It corresponds to the one of the leading causes of death and severe disability in adults worldwide. Ischemic stroke is the most common type of stroke corresponding to 85% of all strokes [1]. Pathophysiology of ischemic stroke involves a complex and dynamic process which is not limited to neurons but involves all brain cells and extracellular matrix (ECM) in a “glioneurovascular niche” that interacts with the peripheral immune system. Stroke patients could benefit from reperfusion therapies up to 6 h after ischemic stroke onset [2]. After these first hours, there is no effective treatment available besides rehabilitation [3].

Development of innovating therapies using stem cells or trophic factors can enhance brain remodeling; this process is crucial and success requires a pathophysiological viewpoint [4]. These approaches also have the advantage of action over an extended therapeutic time-window after stroke and thereby might be effective in more patients than those helped by current acute strategies such as thrombolysis and thrombectomy. Cell-based therapy has been proposed as a potential source of new cells to replace lost cells due to central nervous system injury, as well as a source of trophic molecules to minimize damage and promote recovery [5, 6].

Stem/progenitor cell transplantation improves recovery after stroke in rodent models [7]. Nevertheless, there are two main limits concerning clinical translation in cell transplantation in stroke [8].

**Figure 1:**
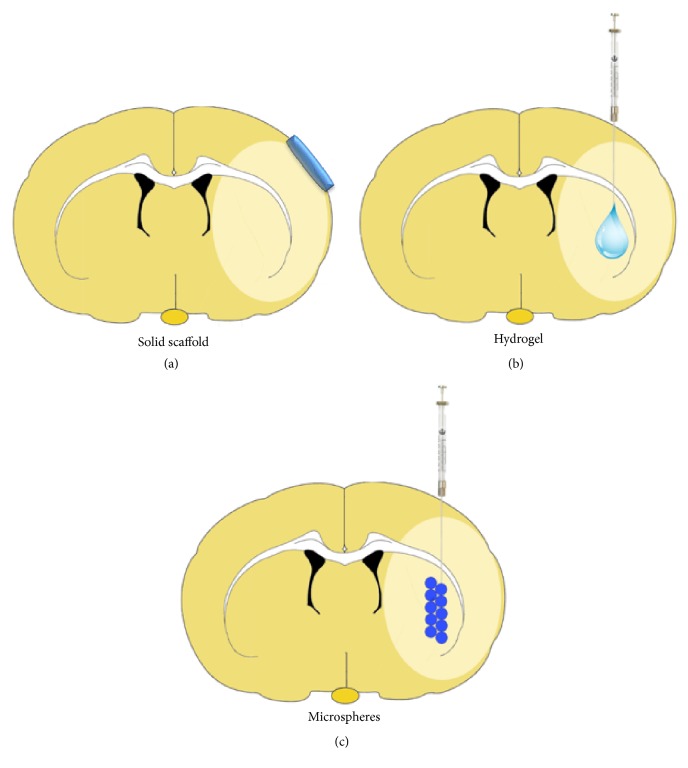
Schematic illustration of different biomaterial applications on ischemic brain. (a) Solid brain scaffolds for surface application and gradual liberation of cells, drugs, or growth factors. (b) Injectable hydrogel, in liquid phase with an* in situ* gelation. (c) Microspheres for gradual intracerebral delivery.

Firstly, when the stem/progenitor cells are systemically administrated, this requires the administration of a high number of cells and only a few amounts of cells achieve the brain [9]. An alternative way is the intracerebral (IC) administration of cells directly into the brain parenchyma and/or into the lesion cavity [10] (Figure 1). This location is a compartmentalized area of lost tissue that has undergone necrosis and can allow a large volume injection, and it is directly adjacent to peri-infarct zone [11], site of greatest neuroplasticity after stroke [12].

The second point concerning cell administration is the important cell death observed after IC graft. After stroke, within the infarct cavity, a very important loss of ECM in addition to neuronal and glial cell loss is noted. This cavity is filled by extracellular fluid and proteins from leakage of plasma proteins [13]. This damaged area is a hostile environment for cell transplantations resulting in a severe loss of grafted cells [14, 15].

Recent advances in tissue engineering have produced applications that may provide solutions to the problem of transplanted cell death and damage associated with the transplant [11]. Biopolymer hydrogels have been projected to promote cell survival and engraftment (Figure 1).

Currently, biomaterials researchers are seeking to optimize injectable hydrogels by combining cell seeding with the incorporation of growth factors or tracers. The use of biomaterials to improve benefit of cell therapy after stroke must be carefully investigated in experimental studies prior to transferring this promising procedure to clinical trials.

In this paper, we aim to review the different applications of biomaterials after ischemic brain lesion and to explore specific features such as the choice of biomaterial compounds, physical and mechanical properties, biocompatibilities, and degradation regarding recent studies in experimental stroke (Table 1).

*Stem Cell in Stroke Repair*. The benefits of exogenous cell-based strategies include their potential to rescue damaged brain tissue by simultaneously promoting endogenous neuroprotection and neural repair (including neurogenesis, angiogenesis, oligodendrogliogenesis, axonal sprouting, and synaptogenesis) [6]. Additionally, these cells could act in synergy with endogenous stem cells. The different cell sources and types were recently reviewed by Jendelová et al. [16].

Currently, we distinguish two main strategies of cell therapy: (1) a paracrine trophic support using “peripheral” stem or stromal cells and (2) a direct neural replacement using neural stem/progenitor cells or mature cells such as neurons. The route, dose, and timing for cell transplantation after stroke are still debated, depending on the chosen cell product and the expected therapeutic effect.

Direct replacement of injured neurons (“homotopic” repair) has been suggested after neural stem cells (NSC) IC administration [17] or intra-arterial (IA) injection [18]. These results were demonstrated by using induced pluripotent stem cells (iPSC) derived neurons [19], bone marrow stromal cells (BMSCs) [20], or embryonic stem cells (ESC) derived mesenchymal stem cells (MSC) injections [21]. However, only a few grafted cells can be expected to express neuronal markers, and long-term graft survival is relatively poor [22–26]. Moreover, despite possible integration of grafted NSCs [27–29] into the host circuitry, functional recovery occurs, too early to be caused by newly formed neurons and synapses.

The effects of cell therapies on poststroke vasculogenesis and angiogenesis seem to be crucial. IC injection of endothelial cells can improve vasculogenesis linked to neurogenesis via vascular endothelial growth factor (VEGF) release mechanisms [30]. Proangiogenic effects were also observed early after MSC injection contributing to VEGF-induced angiogenesis [31], after injection of NSC [32, 33], endothelial progenitor (EP) [34], or cord-blood mononuclear cells CD34+ [35]. Moreover, EP, MSC, or NSC can also facilitate protection or restoration of the blood-brain barrier after stroke [33, 36, 37].

Another important effect of cell therapy is enhancement of glial remodeling and limitations in anterograde degeneration [38–40]. For example, intravenous (IV) injection of MSC has beneficial effects on both poststroke glial remodeling and axonal remyelination [41]. It also increases glial cell-derived neurotrophic factor (GDNF) levels, creating a hospitable environment for neural repair and neuroblast migration from the subventricular zone (SVZ) [42].

Additionally, cell therapies can limit host cell death through antiapoptotic and immunomodulatory mechanisms. Although MSCs are known to attenuate microglia and leukocyte inflammatory responses after stroke [43–45], some immunomodulatory properties were also observed for cord-blood cells [46] or NSC [47, 48], which can both influence splenic inflammatory responses after stroke [49].

## 2. Biomaterials as Cell Scaffold to Enhance Cell Graft

An important cell death is reported after IC graft into the damaged area [14, 15]. The use of “carrier” scaffolds is particularly relevant for injections into the stroke cavity at a chronic stage, avoiding a deleterious injection into the adjacent brain tissue where important recovery processes may be underway.

Enhancing the graft survival after IC injection is the common aim of several ongoing experimental strategies. Advances in regenerative medicine are increasingly providing new opportunities to repair damaged tissue by using biomaterials to enhance cell graft. Biomaterials are materials specially developed for use in tissues with the minimum of biological response to the foreign body. Furthermore, biomaterial seems to improve graft cell survival, proliferation, migration, and differentiation, protecting grafted cells from immune response and thus improving cell therapy effects.

A study using matrix gel scaffolding associated with human ESC neuronal precursor cells (NPCs) administrated 3 weeks after an experimental ischemic stroke in rats demonstrated beneficial effects induced by biomaterial coadministration. The effects include cell survival and neuronal differentiation, reduction of infarct volume, and improvement of functional outcome [50].

Biomaterials improve cell survival even if these cells are administrated in the intact brain adjacent to the lesion. A study using a thermoreversible gelation polymer (TGP) as scaffold in MSCs transplantation demonstrated that the association of MSC-TGP significantly improved cell survival [51]. The fate of transplanted MSC was examined 8 weeks after transplantation with immunohistochemistry. The majority of cells were positive for both NeuN and MAP2 [51].

Zhong et al. tested the effects of a Hyaluronan-Heparin-Collagen based hydrogel in cell protection* in vitro* [11]. Stem cell survival was tested under conditions of growth factor and nutritional support and under conditions of stress induced by growth factor and nutrition withdrawal to mimic the initial transplant state. In stem cell cultures with nutrient and growth factor support, the hydrogel modestly but significantly increased survival. In stem cell cultures without such support, the hydrogel substantially increased the survival [11]. Furthermore, they demonstrated that this hydrogel was able to improve the survival of NPCs into the brain cavity after stroke. Additionally, the authors reported a reduction of inflammatory cells infiltration into the graft. Active microglia/macrophages infiltrating the cell engraftment were significantly decreased with hydrogel [11].

Such as described below (see “Interest of Biomaterials in Cell Therapies”), the inflammatory response is an important step of healing process. Nevertheless, it is recognized that a reduced inflammatory response can result in a more favorable outcome.

Biomaterials alone are able to modulate the inflammatory response. In a cortical brain damage model, a three percent HA gel was coated onto the lesion for the experimental groups and normal saline solutions for the control groups. The results from immunohistological analysis put in evidence a significant reduction of the number of GFAP^+^ cells [52].

The ultimate goal of stroke treatment is the functional recovery. Identifying behavioral deficits in animal models of stroke is essential for potential translational applications [53]. As we noted, regenerative approaches such as cell therapy and administration of trophic factors provide an increase in endogenous brain structural plasticity and motor remapping after ischemia [54]. The use of biomaterials may enhance these functional effects. Emerich et al. have demonstrated that alginate hydrogel used as implant for sustained release of VEGF promotes functional and structural protection from ischemic damage after transient ischemia [55]. The group treated with VEGF-Hydrogel had an important decrease (about 80%) in lesion volume evaluated by 2,3,5-triphenyltetrazolium chloride (TTC) staining. Behavioral analysis using motor asymmetry and neurologic scores demonstrated that recovery is improved by the association of hydrogel-VEGF compared to VEGF alone [55]. Similarly, Guan et al. demonstrated that human MSCs transplanted with collagen scaffolds in a model of brain injury present better outcomes compared to MSC alone [56]. Collagen scaffolds increased the retention of MSC in the lesion site and limited its distribution at the transplanted region resulting in better functional recovery during 4 weeks after transplantation [56]. Another study assessed the combination of NSC and collagen type-I administrated 24 hours after stroke and showed an improvement of the structural and functional recovery [57]. In this study, rats were submitted to a transient ischemia and received a graft of a brain scaffold of collagen type-I seeded with NSC. The evaluation by microscopy showed that, 30 days after transplantation, NSC-collagen group presented new synapses and better functional recovery, while at this time point collagen has been completely degraded [57].

### 2.1. Interest of Biomaterials in Cell Therapies

Some minutes after blood flow interruption and energetic deprivation, a cascade of cellular and molecular mechanisms are activated resulting in cell death.

Inflammation is initiated by necrosis and tissue injury through the recognition of damage associated molecular patterns [58]. The process of activation of inflammatory response is currently incompletely understood [59]. Inflammation subserves a number of biological functions and can have both positive and negative consequences [60]. This process is necessary to remove necrotic and apoptotic cells and cleaved extracellular matrix molecules and to initiate angiogenesis and tissue repair [61]. However, exacerbates and chronic inflammation lead to the formation of inhospitable environment for regeneration and cell grafting, resulting in further cell death.

The ischemic lesion promotes changes in extracellular environment such as ECM. The ECM is a three-dimensional, noncellular structure composed of collagens, elastin, proteoglycans (including hyaluronan), and noncollagenous glycoproteins [62] in healthy conditions. ECM macromolecules are bioactive and modulate cellular events such as adhesion, migration, proliferation, differentiation, and survival [63]. During brain ischemia, the basement membranes of blood-brain barrier are degraded and new ECM proteins are deposited in brain parenchyma, either by secretion from activated glia or by leakage of plasma proteins, such as fibrinogen [64]. The significance and consequences of these changes may vary with the time point after injury [13].

Remodeling and repair of brain parenchyma are influenced by ECM composition. Stroke induces alterations in ECM such as increase of proteoglycans [65], inhibition of neurite outgrowth by astrocytic activation [66], and upregulation of matrix-metalloproteinases (MMPs) [62]. These mechanisms can contribute negatively to endogenous remodeling and to the host response to cell therapy.

To enhance structural and functional recovery after stroke, biomaterials protecting grafted cells and/or supporting repair processes such as ECM substitute are currently in development and could be a promising neurorestorative approach.

## 3. Biomaterials Components

Biomaterials are based on natural or on synthetic compounds used alone or in mixtures, providing a polymer with different properties [67]. The choice of biomaterial is of importance because it can influence biomaterial effectiveness and the response of host tissue. Natural polymers such as hyaluronan, chitosan, and collagen are advantageous because they have already been used in clinical applications as injectable hydrogels such as lubrifiants, wound sealants, viscosupplements, or filling agents in esthetic medicine [68, 69].

On the other hand, synthetic hydrogels can be engineered to more accurately mimic the physical and mechanical characteristics of ECM [70]. The advantage of synthetic biomaterials is the ability to tightly control the polymerization, degradation, and biocompatibility of hydrogel. Synthetic hydrogels are better chemically defined and in most cases are biologically inert, reducing potential immune reaction into the brain [70]. In this section, we explore some components used in recent studies in experimental stroke.

### 3.1. Chitosan

Biomaterial can be produced from chitosan, a natural polysaccharide produced by deacetylation of chitin from crustacean shells [71]. Chitosan-based biomaterials have been used in different applications such as corneal wound healing [72], peripheral nerve injury [73], and mechanical brain injury [74]. In a recent study, chitosan hydrogel coadministrated with ESC-derived endothelial cells showed a positive effect by presenting a high cell survival and minimal cytotoxicity* in vitro* [75]. When this chitosan-based hydrogel encapsulating mixed adult and endothelial cells and containing VEGF was implanted into a mouse model of hindlimb ischemia, it induced neovascularization through vasculogenesis and angiogenesis. It also led to recovery of blood flow in ischemic hindlimbs [75]. Ding et al. have demonstrated a most pronounced neuroprotective effect mediated by acetyl-11-keto-*β*-boswellic acid (AKBA) loaded in O-carboxymethyl chitosan nanoparticles (NPs) when compared to the AKBA only in a rat model of ischemic stroke [76]. The combination AKBA+NPs promoted a functional improvement by reducing infarct volume and apoptosis [76]. However, chitosan-based biomaterials present some disadvantages such as a fast biodegradation* in situ*. Additionally, the compatibility of chitosan with physiological medium depends on the preparation method. Residual proteins could indeed cause allergic reactions [71].

### 3.2. Hyaluronic Acid (HA)

**Figure 2:**
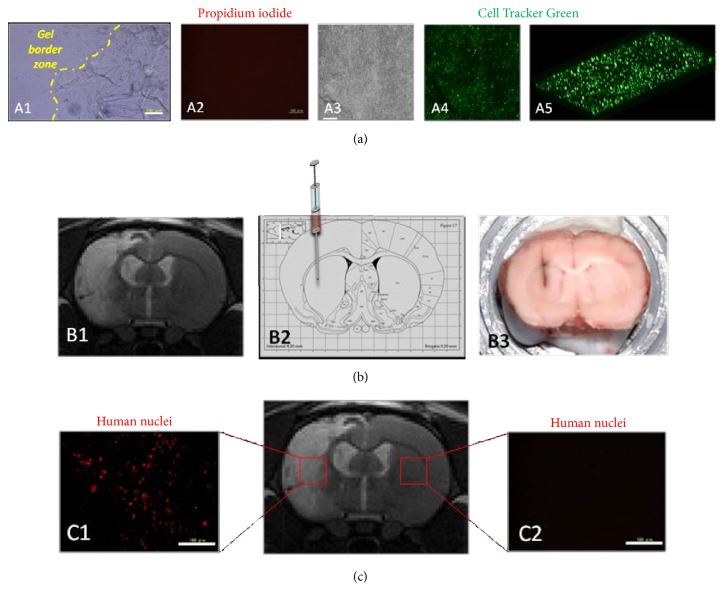
Different experimental steps for intracerebral graft of cell-biomaterial after stroke. Scale bar = 100 *μ*m. (a)* In vitro* biocompatibility: after mixing human mesenchymal stem cells (MSC) within hyaluronic acid (HA) hydrogel (Hystem HP, Sigma: hyaluronan+polyethylene glycol diacrylate), MSC survived into the gel during several days in culture (A1) without cell death (A2, propidium iodide cell dead assay). Cell survival and spreading into the HA gel were assessed in one-week culture (A3) using confocal microscopy and confocal microscopy stacks and viable cell labelling (A4 and A5, Cell Tracker Green CMFDA, Life). (b) Intracerebral transplantation: one week after experimental ischemic stroke in rat, magnetic resonance imaging was used to determine the injection site into the stroke cavity near plastic areas surrounding the lesion (B1). Coordinates for stereotactic injection were defined using anatomic atlas (Watson-Paxinos) (B2). By histology, the stereotactic tract can be macroscopically observed (B3, crysostat section). (c)* In vivo* biocompatibility and effects:* ex vivo* brain immunohistology demonstrated cell survival into the graft site such as human MSC identification in stroke lesion (C1, human-specific monoclonal antibody to nuclear antigen, MAB1281, 1/1,000, Chemicon) without cell migration in contralateral hemisphere (C2). Additional experiments must be done to assess long-term cell differentiations and host integration, hydrogel biodegradation, local inflammatory response, and behavior recovery effects.

Another promising material is HA, an abundant glycosaminoglycan in the brain ECM [13, 62, 63]. HA is a linear polymer composed of the repeating disaccharide unit of D-glucuronic acid and N-acetyl-D-glucosamine. This polysaccharide plays a key role in many biological processes such as stabilizing the ECM, regulating cell adhesion and motility, and mediating cell proliferation and differentiation [77]. Liang et al. reported an increase in engrafted cells' survival and proliferation of three different cell lines (C17.2 cells, human neural progenitor cells (ReNcells), and human glial-restricted precursors) into a HA-gelatin-polyethylene glycol diacrylate (PEGDA) gel, although a mild inflammatory response towards the implanted hydrogel was observed [8]. As an example, we report here that the same HA hydrogel can be used for MSC transplantation after experimental stroke (Figure 2).

### 3.3. Poly(Latic-co-glycolic Acid) (PLGA)

PLGA is one of most commonly used biodegradable synthetic polymers for three-dimensional (3D) scaffolds in tissue engineering [78]. The advantage of synthetic polymers is a high control of degradation rate and mechanical properties [79]. PLGA is biocompatible and has been investigated to increase cell survival. NSCs grafted into PLGA slices of 2 mm depth were viable after 14 days of culture [80]. PLGA can be used to produce microspheres for a gradual delivery of cells or drugs [81–83]. Bible et al. optimized the conditions for cell attachment in order to preserve the MHP36 cell line properties in PLGA microspheres [84]. In this experiment, 100–200 *μ*m PLGA microparticles that were modified with poly(allylamine) via plasma polymerization of allylamine and further coated with plasma-derived fibronectin were administrated into the lesion cavity (two weeks after stroke). They demonstrated a primitive* de novo* tissue formation within 7 days [84]. Another interesting study using PLGA microspheres showed that a single subcutaneous administration of ONO-1301 (a long-acting prostacyclin agonist) in PLGA microspheres was able to improve poststroke recovery, edema, and infarct volume in rats [81].

There are some concerns that PLGA degrades into acidic by-products within the brain that may exacerbate inflammation and secondary damage after brain injuries [79]. The less explored poly-*ε*-caprolactone (PCL) polymers might be a safer alternative. PCL induced a lower inflammatory response than PLGA, as demonstrated by lower activated macrophages and glial fibrillary acidic protein (GFAP) expression [85].

## 4. Mechanical and Physical Properties

Biomaterials can be produced using various types of polymerization and take on distinct forms such as solid scaffolds, hydrogels, or micro/nanoparticles. The choice of biomaterial stiffness depends on the administration site target (brain surface or parenchyma), delay of release intended (gradual or immediate), and the therapeutic goal (Figure 1).

Solid scaffolds require surgery to implant and thus are more suitable for surface application [86, 87].* In situ* gelling hydrogels and particles can typically be delivered in a minimally invasive manner using a syringe without the need of open surgery [58]. Hydrogel micro/nanoparticles are also suited for protein, gene, and drug delivery [76, 88, 89]. Hydrogels can be used to graft cells and to provide a microenvironment that can be tuned, promoting cell survival and improving function [8, 11, 57] (Figure 1).

### 4.1. Brain Scaffolds

In a recent study, Hwang et al. used a model of corticectomy to monitor in a noninvasive way by bioluminescence the behavior of stem cells embedded within poly-L-lactic acid (PLLA) scaffold [86]. Human NSCs expressing enhanced firefly luciferase were implanted into the ablated area with or without a PLLA scaffold. They have demonstrated that NSC survived over 14 days compared with 8 days for the nonencapsulated cells [86]. The mechanical strength or stiffness of a hydrogel is named compressive modulus measured in kPa. Solid scaffolds can be projected to present compressive moduli that stimulate cell survival and proliferation [90].

### 4.2. Injectable Hydrogels

Hydrogels are 3D cross-linked networks of water-soluble polymers [91]. Hydrogel polymers can absorb a high water content up to 99%, due to their hydrophilic nature [70], and can be engineered in a variety of physical forms, including a liquid state for* in situ* cross-linking [8]. They have excellent nutrient and oxygen permeability, allowing cell survival in the scaffold [92]. The most important advantage of this kind of biomaterial is that hydrogels form* in situ* [79], allowing an administration with a minimal invasiveness by injection [8, 11, 68]. The cross-linking process can be induced by temperature [93], pH [94], or addition of a synthetic cross-linker such as PEGDA for HA hydrogel. Besides, hydrogels possess elastic properties that are similar to those of brain tissue. Hydrogels injected in liquid phase usually present low compressive moduli after polymerization, which promotes a stem cell differentiation toward neural lineages [90].

Lam et al. assessed the effect of cell therapy by administrating neural progenitor cells derived from iPSC (iPSC-NPC) into the infarct cavity of mice submitted to a cortical photothrombotic stroke. iPSC-NPCs were encapsulated in a HA hydrogel matrix or in PBS [95]. The combination (hydrogel + iPSC-NPC) was able to promote differentiation of the neural progenitor cells to neuroblasts. Despite this good result, it did not increase cell survival one week after transplantation [95]. The hydrogels used in this study were synthesized to contain the adhesion peptide and were cross-linked with either matrix-metalloproteinase (MMP) degradable peptides or non-MMP degradable peptides. The hydrogel was specifically engineered to have a compressive modulus of ~3 kPa because that is the approximate stiffness of the brain [96]. In a recent study, Massensini et al. assessed rheological properties and gelation at body temperature of a biological hydrogel produced from porcine urinary bladder ECM [97]. They performed an efficient MRI-guided injection with drainage of fluid from the cavity to assess* in situ* hydrogel formation and ECM retention at different concentrations. The concentrations superior than 3 mg/mL polymerized within stroke cavity, whereas lower concentrations remained in liquid phase permeating the peri-infarct area.

A downside to hydrogels is that cell migration and outgrowth are often poor due to its weak mechanical structure [79]. Moreover, biodegradation rate is hard to control [98] and must be carefully investigated in the future (see Section 5).

### 4.3. Microencapsulation

Biomaterials can also be used to encapsulate molecules, cells, cell aggregates, or drugs with the aim of promoting a gradual liberation [88, 99] or graft protection. Molecules or cells encapsulation can be automatized to provide a large number of implantable “active” capsules. This could be an alternative to intracerebroventricular injection through the catheter/osmotic minipump systems. This strategy provides a gradual release and a sufficient penetration of growth factors in brain tissue [99]. Nakaguchi et al. have demonstrated an increase in the endogenous neurogenesis in the SVZ of adult mice induced by IC administration of growth factors: insulin-like grow factor 1 and hepatocyte growth factor encapsulated into gelatin hydrogel microspheres [100]. For NSC grafting, Skop et al. optimized multifunctional and biocompatible chitosan-based films and microspheres. Heparin was covalently cross-linked to the chitosan scaffolds which bound fibroblast growth factor-2 (FGF-2) and sustain survival and growth of NSC [101].

### 4.4. Neural Networks as Potential Strategy

In general way, cell replacement strategies do not take account of the complexity of the brain. Indeed, the cerebral abilities are linked to highly complex connections established between specialized neuroanatomical regions. Replacing lost neurons and extracellular environment does not warrant the restoration of this complex network of axonal tracts. Focusing on this question, alternative biomaterials have been developed with the aim to restore long-distance axonal connections. Replacing lost neurons and extracellular environment does not warrant the restoration of this complex network of axonal tracts. Focusing on this question, alternative biomaterials have been developed with the aim of restoring long-distance axonal connections.

A recent strategy in neural tissue engineering involves the development and application of “living scaffolds,” which are defined as constructs with a controlled, often heterogeneous, and anisotropic 3D cell architecture and biomaterial composition [102]. This living cellular-biomaterial scaffold presents a new form to implant biomaterials and cells. These living scaffolds are able to orientate, give support to, and aid regenerating cells and/or processes (e.g., axons), mimicking crucial aspects of developmental path finding [102]. The cells constitute the “living” component of scaffolds.

A very interesting study of Struzyna et al. [103] using microtissue engineering neural networks for reconstituting the architecture of axonal tracts demonstrates that this approach is effective in promoting survival at least one month and additionally they detected neurite penetration and synapse formation [103]. In this referred study, the microtube was constructed based on an agarose hydrogel and the interior containing extracellular matrix proteins and cerebral cortical neurons and was implanted in healthy rat brain. This very encouraging result presents a great potential for neuroregenerative therapy and may ultimately facilitate functional recovery if it could be transposed/overlapped in stroke models in the future.

## 5. Biomaterials Degradation

Many materials formulated for tissue engineering and/or the release of therapeutics are designed to be biodegradable (or bioresorbable) to reduce the complications of tissue scarring and glia tumor formation from permanent implants [70]. Thus, it is necessary to determine the biodegradability of materials* in vitro* and* in vivo* [104]. Independent of their composition (cross-linking reagents and the functional group of HA derivatives), HA hydrogels have variable degradation rate. By example, in Hahn et al., the authors demonstrated that HA hydrogels prepared with three different cross-linking reagents have variable degradation test results [105]. Indeed, adipic acid dihydrazide grafted HA (HA-ADH), methacrylated HA (HA-MA), thiolated HA (HA-SH) were compared and according to* in vitro* degradation tests, HA-SH hydrogel was degraded very fast, compared to HA-ADH and HA-MA hydrogels and HA-ADH hydrogel was degraded slightly faster than HA-MA hydrogel. Moreover, when HA-MA hydrogels and HA-SH hydrogels are implanted in the back of rats, HA-SH hydrogel was* in vivo* degraded completely only in 2 weeks, whereas HA-MA hydrogels were degraded only partially even in 29 days. There was no adverse effect during the* in vivo* tests.

## 6. Biocompatibilities with Therapeutic Cells and Host Tissue

Brain is mostly isolated from the periphery by the blood-barrier. It has a similar but slightly different response to tissue damage and foreign materials [70]. The use of biomaterials, such as hydrogels, as neural cell delivery devices is becoming more common in areas of research such as stroke, traumatic brain injury, and spinal cord injury.

When reviewing the available research, there is some ambiguity in the type of materials used and results are often at odds. Hydrogels must be designed to be biocompatible with the implanted cells [106] and with the tissue environment.* In vitro* cultures of embedded cells to assess cell compatibility and functionality must be done prior to* in vivo* graft (Figure 1). 3D cultures can be useful to precise cell location and cell distribution into a gel.

*In vivo*, a wide variety of synthetic polymers have been shown to be biocompatible in the body, such as polyesters and acrylates [107–110]. Natural polymers, such as poly(amino acids) and HA, have been modified to form biocompatible hydrogels. Polymeric hydrogels placed into a fimbria-fornix lesion cavity promote fiber (re)growth in morphological study in the rat [111–114]. This biocompatibility refers to the histocompatibility of an implanted hydrogel and the local and systemic response of the host which includes the inflammatory and immune reaction of the brain [70]. Implanted biomaterials promote a foreign body response. This inflammatory response presents a variable level which varies depending on the material choice and the site of implantation [115]. After implantation, a biomaterial acquires a layer of host proteins that is associated with the surface chemistry of material [58].

Brain tissue engineering in the postinjury brain represents a promising option for cell replacement and rescue, providing a cell scaffold for transplanted or resident cells. But a number of natural biomaterials have intrinsic anti-inflammatory properties, including HA and chitosan [116]. Thus, they are suitable as carriers for anti-inflammatory therapeutics. However, synthetic materials are also capable of acting in an anti-inflammatory way. Zhong et al. have demonstrated a beneficial effect of a hyaluronan-heparin-collagen hydrogel by promoting the survival of ES-NPCs and by reducing inflammatory infiltration of the graft with the hydrogel transplant [11]. However, further optimization of hydrogel compositions is warranted to avoid possible inflammatory responses such as those observed in immunocompetent mouse brain 2 weeks after IC injection of a HA hydrogel preseeded with human NSCs or glial precursors [8]. Indeed, HA degradation is facilitated in inflammation and injury by the production of reactive oxygen and nitrogen species [77, 117]. HA is degraded* in vivo* by hyaluronidases (HAases) into shorter fragments. However, the extent of HA degradation that occurs under pathological conditions may be greatly enhanced.

## 7. Imaging of Biomaterials Engraftment

Clinical studies can benefit from noninvasive methods to assess brain stroke. Experimental studies also have used noninvasive imaging techniques to monitor grafted cells distribution and their effects on brain tissue [118]. Several imaging techniques such as MRI [31], positron emission tomography (PET) [119], and nuclear imaging [9] have been used to track transplanted cells* in vivo.* Imaging modalities with precise anatomical information like MRI can be used to evaluate the lesion size and extension and to precisely guide biomaterial administration. Furthermore, recent advances using multiparametric MRI enable longitudinal monitoring of vascular remodeling [7, 120] and brain function by using functional MRI.

Bible et al., for example, have demonstrated that NSCs coadministrated with ECM bioscaffold produced from porcine brain and urinary bladder promote the formation of* de novo* tissue in the lesion cavity and repair processes after ischemic stroke evaluated by MRI [121]. Noninvasive imaging by MRI was used to guide the administration of biomaterials in a similar study [97].

**Figure 3:**
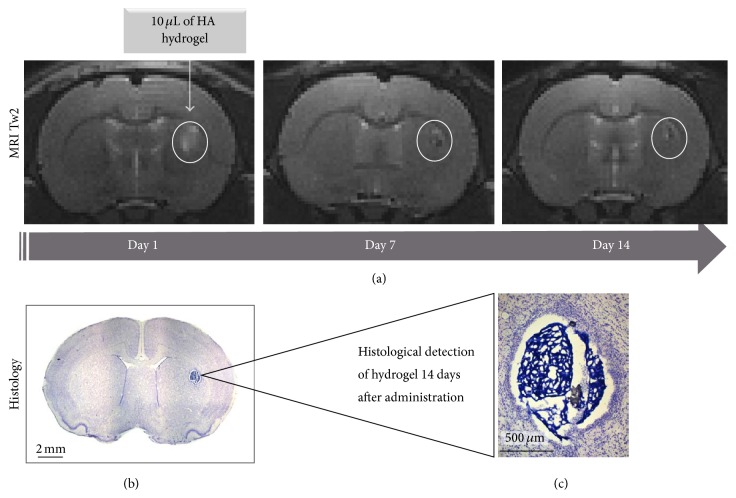
Representative images of* in vivo *and* ex vivo *detection of hyaluronan-acid (HA) hydrogel. (a)Magnetic Resonance Imaging (MRI) weighed in T2, hydrogel detected at different time points (days one, seven, and fourteen after administration). (b) Cresyl violet staining of HA hydrogel acquired two weeks after administration, noted in (×2) (b) and (c) (x10) magnification. These images demonstrate efficient local gel formation instead of liquid diffusion which would be due to a delayed polymerization after infusion and the* in vivo *stability of HA hydrogel.

Noninvasive evaluations are a powerful tool for determining the efficacy of the combination biomaterials with cell therapy, allowing a validation of biomaterial application by a correlation of* in vivo* images and histological findings (Figure 3).

## 8. Conclusion

The use of biomaterials for stroke therapy provides a promising avenue for cell transplantation, especially in the brain where the regenerative properties can be limited.

However, the choice of biomaterial compounds and properties must be adapted, according to biocompatibilities with embedded cells and host tissue and to biodegradation rate. Thus, collaborations between bioengineering researchers and neuroscientists are required to validate and optimize preclinical experiments. Purification of biomaterials is imperative for safe use in humans. Therefore, it requires rigorous tests of cytotoxicity. Important aspects such as reproducibility, correlation with behavioral outcomes, and a long-term assessment of biomaterials degradation should be considered before clinical translation.

Hydrogels could be used to enhance cell transplantation benefit in patients by stereotactic injection of liquid hydrogel with* in situ* polymerization, or by surgical graft of stiffer cell-biomaterial layers, for example, during a hemicraniectomy for large stroke or during subsequent reparative cranioplasty. An appropriate follow-up with a noninvasive brain imaging to track hydrogel degradation and brain remodeling is strongly indicated.

## Figures and Tables

**Table 1:** Examples of biomaterials applications in experimental stroke.

Cells/growth factors	Species/stroke model	Biomaterial	Outcomes	References
hRecombinant osteopontin	Rats tMCAO	Gelatin type A microspheres	↓ of infarct volume neurological deficits	Jin et al. 2014 [122]

rBMSCs	Rats pMCAO	N-Isopropyl- acrylamide polymer sheets	Improvement of motor function	Ito et al. 2014 [87]

Pegylated EGF and EPO	Mice focal ischemia endothelin-1	PEG microparticles PLGA nanoparticles dispersed in a (HAMC) hydrogel	↓ of inflammation, ↓ of infarct volume	Wang et al. 2013 [99]

hNSC	Rats tMCAO	VEGF-PLGA microparticles	Neovascularization, angiogenesis	Bible et al. 2012 [123]

iPS-NPCs	Mice cortical photothrombotic	HA, acrylate	↑ of differentiation to neuroblast	Lam et al. 2014 [95]

hNSC	Rats tMCAO	Xenogeneic (ECM) bioscaffold	Formation of *de novo* tissue	Bible et al. 2012 [121]

ONO-1301	Rats tMCAO	Subcutaneous (PLGA) microspheres	Neuroprotection and ↓ side effects compared to OA	Hazekawa et al. 2012 [81]

HMGB1	Rats tMCAO	Gelatin microspheres	↓ infarct volume	Jin et al. 2011 [124]

EGF	Mice focal ischemia endothelin-1	PEG microparticles dispersed in a (HAMC) hydrogel	↑ neural stem/progenitor cells	Cooke et al. 2011 [125]

NSC	Rats tMCAO	Collagen type I matrix	↑ synapses and functional recovery	Yu et al. 2010 [57]

hVEGF	Rats tMCAO	Alginate hydrogel	↓ infarct volume ↓ functional deficits	Emerich et al. 2010 [55]

MCAo p or t, permanent or transient middle cerebral artery occlusion; BMSCs, bone marrow stromal cells; EGF, epidermal growth factor; EPO, erythropoietin; SC, stem cells; PEG, polyethylene glycol; HAMC, hyaluronan methylcellulose; h, human; NSC, neural stem cells; VEGF, vascular endothelial growth factor, iPS, induced pluripotent stem; HA, hyaluronic acid; NPCs, neural pluripotent cells; PLGA, poly lactic-co-glycolic acid; OA, oral administration; HMGB1, high-mobility group box 1 protein; ECM, extracellular matrix.

## Abbreviations

ADH: Adipic acid dihydrazide
AKBA: Acetyl-11-keto-*β* boswellic acid
BBB: Blood-brain barrier
BMSC: Bone marrow stromal cells
CCL-2: Chemokine ligand 2
ECM: Extracellular matrix
EGF: Epidermal growth factor
EP: Endothelial progenitor
EPO: Erythropoietin
ESC: Embryonic stem cells
FGF-2: Fibroblast growth factor type 2
GDNF: Glial cell-derived neurotrophic factor
GFAP: Glial fibrillary acidic protein
h: Human
HA: Hyaluronic acid
HAMC: Hyaluronan methylcellulose
HGF: Hepatocyte growth factor
HMGB1: High-mobility group box 1 protein
HMW: High molecular weight
IA: Intra-arterial
IC: Intracerebral
IGF1: Insulin-like growth factor 1
IL-12: Interleukin-12
iNOS: Inducible nitric oxide synthase
iPSC: Induced pluripotent stem cells
IV: Intravenous
kPA: Kilopascals
LMW: Low molecular weight
MA: Methacrylated
MCAo: Middle cerebral artery occlusion
MIP-1*α*: Macrophage inflammatory protein
MSC: Mesenchymal stem cells
NPC: Neural progenitor cell
NSC: Neural stem cells
PBS: Phosphate buffered saline
PEG: Polyethylene glycol
PEGDA: Polyethylene glycol diacrylate
PLC: Poly-*ε*-caprolactone
PLD: Poly-D-lysine
PLLA: Poly-L-lactic acid
SC: Stem cells
SH: Thiolated
SVZ: Subventricular zone
TNF-*α*: Tumoral necrosis factor
TTC: 2,3,5-Triphenyltetrazolium chloride
VEGF: Vascular endothelial growth factor.
